# Supplementary material for: The Role of Potassium and Calcium Currents in the Bistable Firing Transition
Source: bioRxiv. 2023 Aug 17:2023.08.16.553625. Preprint. [Version 1] doi: 10.1101/2023.08.16.553625 (PMC10462112; doi:10.1101/2023.08.16.553625)
Supplement: 1 [file NIHPP2023.08.16.553625v1-supplement-1.pdf]

bioRxiv preprint doi: https://doi.org/10.1101/2023.08.16.553625; this version posted August 17, 2023. The copyright holder for this preprint (which was not certified by peer review) is the author/funder, who has granted bioRxiv a license to display the preprint in perpetuity. It is made available under aCC-BY 4.0 International license.

Supplementary Material

S1 Neuron dynamics

Figure S1 illustrates the biophysical properties of neurons and their dependence on the ionic conductance of the slow potassium M-current with the additional presence of calcium currents. The frequency of action potentials increases with the input amplitude, as shown by the three exemplar voltage traces (Fig. S1A). For input amplitudes of $I = 40$ pA, the neuron presents a single spike after ≈ 150 ms from the start of stimulus (Fig. S1B). The second spike occurs for $I = 47$ pA, where the first frequency F is obtained by $1/\mathrm{ISI}_1$. Regular spiking behavior is observed for $I > 160$ pA (Fig. S1C). In Fig. S1D we show the minimum value of I where $F > 0$ for $g_\mathrm{T} = g_\mathrm{L}$ = 0 (gray circles), the black line is a curve fit

$$I = 82.394 + 1559.2 * g_\mathrm{M} + 1837.3 * (g_\mathrm{M})^2. \tag{20}$$

For g_T = 0.4 mS/cm^2 and g_L = 0.2 mS/cm^2 (orange squares) the fitted curve is

$$I = 74.655 + 1672.5 * g_\mathrm{M} + 1719.6 * (g_\mathrm{M})^2. \tag{21}$$

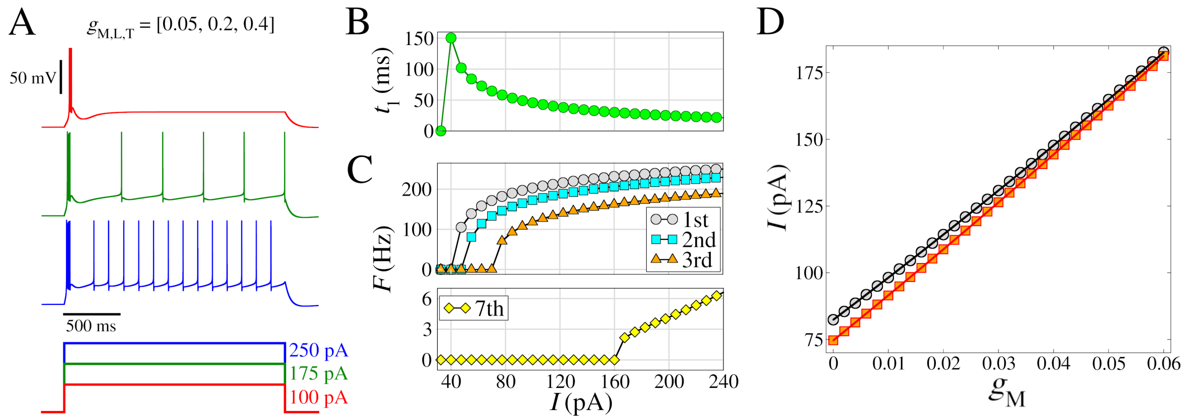

Figure S1: **Model of regular spiking neuron, with I_M, I_T, and I_L. (A)** (Top) Voltage traces with different amplitudes of the depolarizing pulses (bottom). **(B)** Time to first spike (t_1) as a function of the injected current (amplitude of the pulse I). **(C)** Frequency-current curves (F/I), where the instantaneous firing rate (inverse of the inter-spike interval) is represented as a function of I. The curves indicated by different colors correspond to the 1st, the 2nd, the 3rd, and the 7th spike in the train. **(D)** Transition where $F > 0$ for g_T = 0 and g_L = 0 (black line) and g_T = 0.4 mS/cm^2 and g_L = 0.2 mS/cm^2 (red line). Other parameters are $L = d = 96.0$ μm, $g_{leak} = 0.01$ mS/cm^2, E_{leak} = -85.0 mV, g_{Na} = 50 mS/cm^2, $V_T = -55.0$ mV, $g_{Kd} = 5$ mS/cm^2, τ_max = 1000 ms, and g_M = 0.05 mS/cm^2.

Figure S2 presents colored $(g_\mathrm{M}, g_\mathrm{L})$-diagrams for F and CV when I = 200 pA (A–B) and I = 300 pA (C–D). The region in which sustained bursts occur (CV > 1.0) is lower for I = 300 pA than for I = 200 pA. The firing rate (F) increases when I = 300 pA for all $(g_\mathrm{M}, g_\mathrm{L})$-diagram.

bioRxiv preprint doi: https://doi.org/10.1101/2023.08.16.553625; this version posted August 17, 2023. The copyright holder for this preprint (which was not certified by peer review) is the author/funder, who has granted bioRxiv a license to display the preprint in perpetuity. It is made available under aCC-BY 4.0 International license.

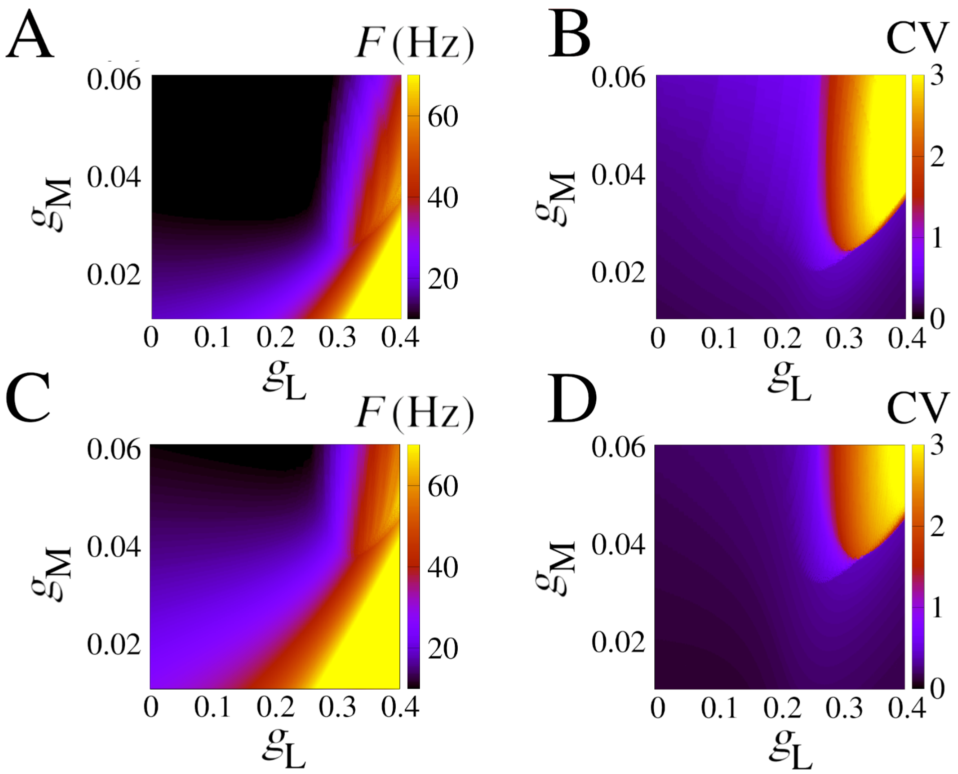

Figure S2: **Firing pattern for diferent I_M and I_L conductances**. **(A)** Firing rate in colored $(g_{\mathrm{M}}, g_{\mathrm{L}})$-diagram for I = 200 pA. **(B)** The same as **A** for the CV. **(C)** Firing rate in colored $(g_{\mathrm{M}}, g_{\mathrm{L}})$-diagram for I = 300 pA. **(D)** The same as **C** for the CV. Other parameters are the same as Fig. S1 with $g_{\mathrm{T}} = 0.4$ mS/cm^2.

S2 Neuron network

In Figure S3 the values of CV, F, and R are shown at the top, middle, and bottom, respectively, in dependence on the constant current and the chemical synaptic conductance (I,g_{syn}) with respect to different values of $g_{\mathrm{M,L,T}}$. The bistable parameter region is identified in white and separates the burst-synchronized region from the asynchronous ones. The high-threshold calcium conductance (g_{L}) allows for transition at lower values of g_{syn} (Fig. S3C). Moreover, the burst-synchronized area is bigger in this case. In contrast, low-threshold calcium promotes the opposite effect by slightly increasing the value of g_{syn} necessary to observe a transition (compare Fig. S3A to B and C to D).

bioRxiv preprint doi: https://doi.org/10.1101/2023.08.16.553625; this version posted August 17, 2023. The copyright holder for this preprint (which was not certified by peer review) is the author/funder, who has granted bioRxiv a license to display the preprint in perpetuity. It is made available under aCC-BY 4.0 International license.

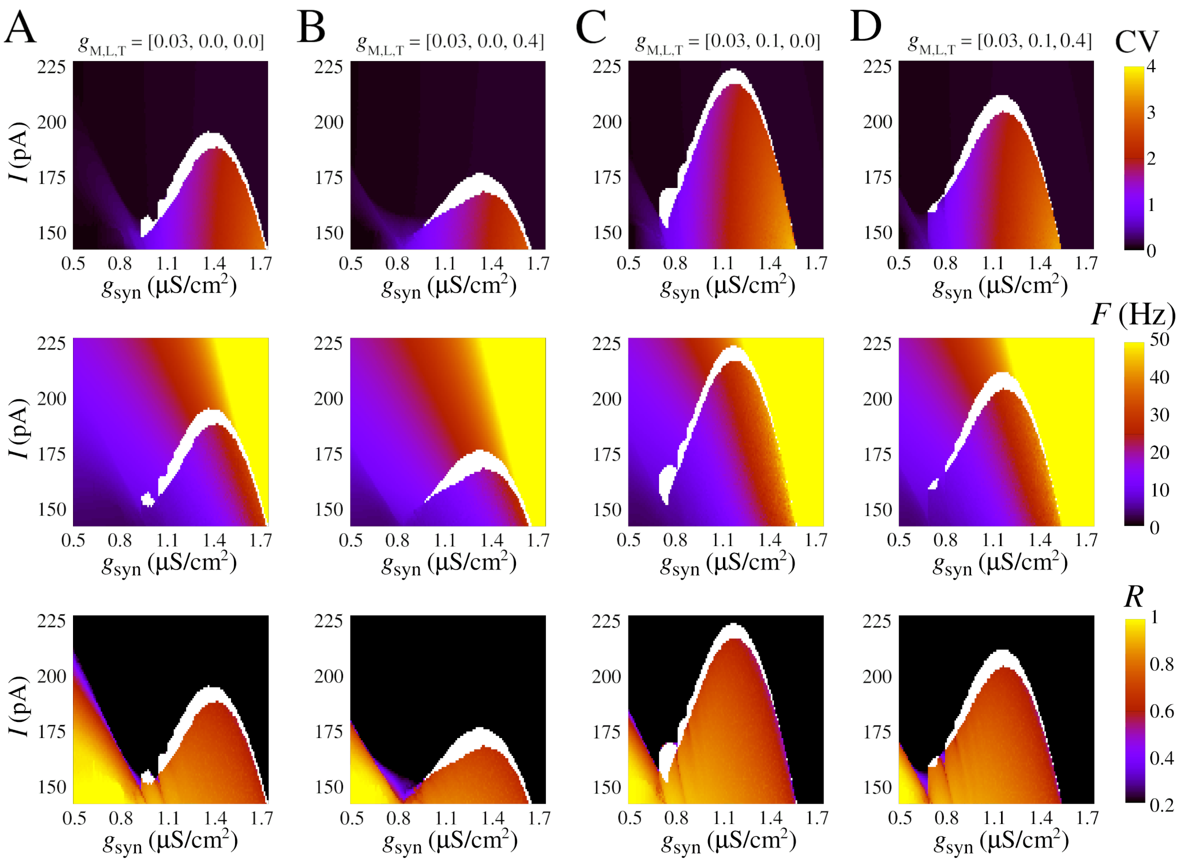

Figure S3: **Changes in network firing pattern with input current (I) and chemical synaptic conductance (g_{syn}).** **(A-D)** CV (top), F (middle), and R (bottom) for different combinations of $g_{\mathrm{M,L,T}}$ (see values atop).
